# Experimental Investigation of Air Compliance Effect on Measurement of Mechanical Properties of Blood Sample Flowing in Microfluidic Channels

**DOI:** 10.3390/mi11050460

**Published:** 2020-04-28

**Authors:** Yang Jun Kang

**Affiliations:** Department of Mechanical Engineering, Chosun University, 309 Pilmun-daero, Dong-gu, Gwangju 61452, Korea; yjkang2011@chosun.ac.kr; Tel.: +82-62-230-7052; Fax: +82-62-230-7055

**Keywords:** air compliance effect, RBC aggregation, blood viscosity, blood viscoelasticity, blood velocity fields, interface in co-flowing streams, microfluidic device

## Abstract

Air compliance has been used effectively to stabilize fluidic instability resulting from a syringe pump. It has also been employed to measure blood viscosity under constant shearing flows. However, due to a longer time delay, it is difficult to quantify the aggregation of red blood cells (RBCs) or blood viscoelasticity. To quantify the mechanical properties of blood samples (blood viscosity, RBC aggregation, and viscoelasticity) effectively, it is necessary to quantify contributions of air compliance to dynamic blood flows in microfluidic channels. In this study, the effect of air compliance on measurement of blood mechanical properties was experimentally quantified with respect to the air cavity in two driving syringes. Under periodic on–off blood flows, three mechanical properties of blood samples were sequentially obtained by quantifying microscopic image intensity (*<I>*) and interface (*α*) in a co-flowing channel. Based on a differential equation derived with a fluid circuit model, the time constant was obtained by analyzing the temporal variations of *β* = 1/(1–*α*). According to experimental results, the time constant significantly decreased by securing the air cavity in a reference fluid syringe (~0.1 mL). However, the time constant increased substantially by securing the air cavity in a blood sample syringe (~0.1 mL). Given that the air cavity in the blood sample syringe significantly contributed to delaying transient behaviors of blood flows, it hindered the quantification of RBC aggregation and blood viscoelasticity. In addition, it was impossible to obtain the viscosity and time constant when the blood flow rate was not available. Thus, to measure the three aforementioned mechanical properties of blood samples effectively, the air cavity in the blood sample syringe must be minimized (*V_air, R_* = 0). Concerning the air cavity in the reference fluid syringe, it must be sufficiently secured about *V_air, R_* = 0.1 mL for regulating fluidic instability because it does not affect dynamic blood flows.

## 1. Introduction

A blood sample is composed of cells (i.e., red blood cells (RBCs), white blood cells, and platelets) and plasma. Given that the number of RBCs is much larger than that of the other cells, RBCs have a significant role in determining mechanical properties of blood samples (viscosity, deformability, and aggregation). In addition, plasma proteins substantially contribute to increasing aggregation. Since a strong relationship between cardiovascular diseases and mechanical properties of blood samples was reported [1,2,3,4], mechanical properties of blood samples have been suggested as label-free biomarkers for early detection of cardiovascular diseases.

In contrast with bulky viscometers [5,6], a microfluidic-based device can provide numerous advantages including fast response, small volume consumption, and disposability. Currently, such devices are widely employed for quantifying mechanical properties of blood samples (viscosity [7,8,9,10], RBC aggregation [11,12,13,14], RBC deformability [3,15], and hematocrit (Hct) [16,17,18]). A microfluidic device has been also employed to separate RBCs or tumor cells from whole blood sample [19,20,21].

In microfluidic environments, blood flows must remain unchanged over time to measure blood viscosity accurately. However, during the process of supplying a blood sample into a microfluidic device, the syringe pump causes fluidic instability at low flow rates [22]. To stabilize unstable flows resulting from the syringe pump, several techniques including air cavity in a driving syringe [23] or microfluidic channel [24,25], portable air cavity unit [22,26], and flexible compliance unit [27,28,29,30,31,32], were demonstrated in microfluidic systems. These methods act on the compliance element in the fluidic circuit model. The compliance was defined as *C* = *ΔV*/*ΔP*. Here, *ΔV* and *ΔP* represents variations of volume and pressure, respectively. This contributes to regulating alternating components and increasing the time constant. Thus, they contribute to removing alternating components of blood flows. Owing to the compliance effect, the fluidic flow remains constant over time. Additionally, the compliance effect tends to delay transient blood flows significantly. Among the aforementioned methods, an air cavity secured inside the syringe is simple and effective because it does not require additional devices. In other words, a disposable syringe (~1 mL) is partially filled with a blood sample (~lower layer) and an air cavity (~upper layer), respectively. Then, the syringe is placed into a syringe pump. Given that the air cavity secured inside the syringe contributes to damping out fluidic fluctuations resulting from the syringe pump [22], the blood flows remain constant over time in the microfluidic channel.

Blood viscosity is measured with a microfluidic device under constant shearing flow condition. After a blood sample and a reference fluid are infused into the microfluidic device at the same flow rate, blood viscosity can be quantified by monitoring the interface in co-flowing streams [8,10]. RBC aggregation is obtained by quantifying the microscopic image intensity of the blood sample under periodic on-off fashion or transient fluidic flows [33,34]. Owing to RBC aggregation in blood samples, the image intensity tends to increase after the blood flow stops suddenly. However, the air cavity secured inside the disposable syringe (i.e., air compliance) has an influence on transient behaviors of blood flows. When turning the syringe pump off suddenly, it takes a longer time to stop blood flows because of the air-compliance effect. Within a specific duration, the blood flow rate does not decrease to sufficiently lower shear rates where RBC aggregation occurs. Thus, it is impossible to quantify RBC aggregation. A simple method to resolve this issue is a pinch valve to stop blood flows immediately [35]. A time constant defining blood viscoelasticity can be obtained by monitoring the interface in co-flowing streams under periodic transient blood flows [33,36]. In other words, after turning the syringe pump off, a time constant is obtained by analyzing the transient behavior of blood flows (i.e., blood velocity). However, when a pinch valve is used to stop blood flows immediately, it is impossible to obtain the time constant throughout transient variations of blood flows. Although air compliance is used effectively for measuring blood viscosity, it hinders the quantification of RBC aggregation or blood viscoelasticity. To quantify the mechanical properties of blood samples (i.e., blood viscosity, RBC aggregation, and viscoelasticity) effectively, it is necessary to quantify contributions of air compliance to dynamic blood flows in microfluidic channels.

In this study, the air compliance effect on measurement of blood mechanical properties was quantified experimentally with respect to the air cavity in two driving syringes. To measure the three aforementioned mechanical properties of the blood sample (i.e., blood viscosity, RBC aggregation, and viscoelasticity), both the blood sample and the reference fluid were filled with individual syringes. The air cavity inside each syringe set a certain volume ranging from 0 to 0.2 mL. After placing them into syringe pumps, the blood sample and reference fluid were infused into a microfluidic channel. The flow rate of the reference fluid remained constant over time. The flow rate of the blood sample was controlled by turning the syringe pump on or off periodically. Three mechanical properties of the blood sample were obtained sequentially by quantifying the microscopic image intensity of the blood sample and the interface between the two fluids flowing in a co-flowing channel, respectively.

As a demonstration, the time constants of test fluids (i.e., blood sample and glycerin (20%)) were first obtained by varying the air cavity in the reference fluid syringe for up to 0.2 mL. Second, variations of the time constant were obtained with respect to Hct = 30%, 40%, and 50%. Finally, to stimulate RBC aggregation, a blood sample (Hct = 50%) was prepared by adding normal RBCs into different diluents (i.e., 1× PBS (phosphate-buffered saline), plasma, and two dextran solutions). The effect of air compliance on the three mechanical properties of blood samples considered was evaluated by varying the air cavity secured in each syringe (~0.1 mL).

## 2. Materials and Methods

### 2.1. Blood Sample Preparation

The Ethics Committee of Chosun University Hospital (CHOSUN 2018-05-11) approved all experimental protocols conducted for this study. Such protocols were deemed appropriate and humane. Gwangju–Chonnam blood bank (Gwangju, Korea) provided concentrated RBCs and fresh frozen plasma (FFP). After conducting washing procedures with a centrifuge twice, pure RBCs were collected from the concentrated RBCs. Additionally, plasma was prepared by thawing FFP at a room temperature of 25 °C. To evaluate the three considered mechanical properties of blood sample with respect to air cavity, various blood samples were prepared by changing hematocrit or diluent. First, to evaluate the contributions of hematocrit or diluent to the time constant (or viscoelasticity), the hematocrit of blood sample was adjusted to Hct = 30%, 40%, and 50% by adding normal RBCs into the diluent (i.e., 1× phosphate-buffered saline (PBS), and plasma). Second, to stimulate RBC aggregation in blood samples, a specific concentration of dextran solution was added into 1× PBS. Dextran powder (*Leuconostoc* spp., MW = 450–650 kDa, Sigma–Aldrich, St. Louis, MO, USA) was diluted with 1× PBS. A blood sample (Hct = 50%) was prepared by adding normal RBCs into two dextran solutions (5, and 10 mg/mL).

### 2.2. Microfluidic Device and Experimental Setup

**Figure 1:**
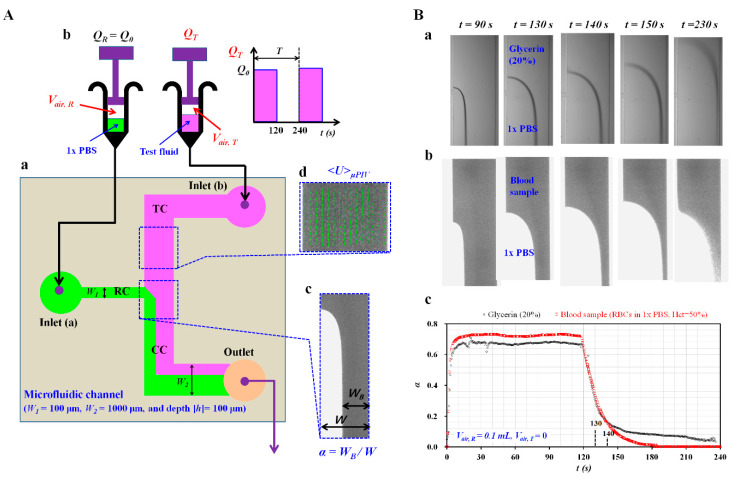
Proposed method for measuring the effect of air compliance on measurement of mechanical properties of blood samples flowing in microfluidic channels. (**A**) Schematic diagram of experimental setup including a microfluidic device, two syringe pumps, and an image acquisition system. (a) A microfluidic device comprising two inlets (a, b), one outlet, two guiding channels for two fluids (reference channel (RC), test channel (TC)), and a co-flowing channel (CC). (b) Two syringe pumps for delivering reference fluid and test fluid. (c) The interface in the co-flowing channel was quantified as *α* = *W_B_*_/_*W*. (d) Velocity fields of blood flows obtained with a micro-particle image velocimetry (PIV) technique. (**B**) As a preliminary demonstration, a blood sample (normal red blood cells (RBCs) in 1× phosphate-buffered saline (PBS), hematocrit (Hct) = 50%) and glycerin (20%) as a test fluid were prepared to show temporal variations of *α*. (a) Microscopic images of a blood sample and 1× PBS flowing in the co-flowing channel at specific time instants (*t* = 90, 130, 140, 150, and 230 s). (b) Microscopic images of glycerin (20%) and 1× PBS flowing in the co-flowing channel at specific time instants (*t* = 90, 130, 140, 150, and 230 s). (c) Temporal variations of α with respect to the blood sample and glycerin (20%).

As shown in Figure 1A-a, a microfluidic device consisted of two inlets (a, b), one outlet, and two guiding channels (test fluid channel (TC, width (*W*) = 1000 μm), reference fluid channel (RC, width (*W*) = 100 μm)), and co-flowing channel (CC, width (*W*) = 1000 μm). To increase blood volume flowing in test channel, channel depth was set to *h* = 100 μm if available under micro-electromechanical-system technique (MEMS) fabrication. A four-inch sized silicon mold was fabricated using the MEMS techniques (i.e., photolithography and deep reactive ion etching). Polymer PDMS (polydimethylsiloxane) (Sylgard 184, Dow Corning, Midland, MI, USA) and a curing agent were mixed at a ratio of 10:1. Mixed PDMS was poured onto the silicon mold. After letting it solidify into a convective oven (70 °C for 1 h), the cured PDMS was peeled off from the mold. Two inlets (a, b) and one outlet were punched with a biopsy punch (outer diameter = 0.5 mm). Using an oxygen plasma system (CUTE-MPR, Femto Science Co., Hwaseong-si, South Korea), the PDMS block was strongly bonded to the glass slide.

To squeeze out the air bubble inside the microfluidic channels and avoid adherence of RBCs to the channels, microfluidic channels were filled with a bovine serum albumin of 2 mg/mL through the outlet. After an elapse of 10 min, the microfluidic channels were filled again with 1× PBS. As shown in Figure 1A-b, a reference fluid syringe was filled with 1× PBS (~0.5 mL) and air cavity (*V_air, R_*), respectively. Likewise, a test fluid syringe was filled with a test fluid (~0.5 mL) and air cavity (*V_air, T_*), respectively. To supply the reference fluid into the reference fluid channel, a polyethylene tubing (length = 300 mm, inner diameter = 0.25 mm, and thickness= 0.25 mm) was connected from a syringe needle to inlet (a). To infuse the test fluid into the blood sample channel, a polyethylene tubing (length = 300 mm, inner diameter = 0.25 mm, and thickness = 0.25 mm) was connected from a syringe needle to inlet (b). To collect both samples from the co-flowing channel, a polyethylene tubing (length = 200 mm, inner diameter = 0.25 mm, and thickness = 0.25 mm) was fitted tightly into the outlet. Using two syringe pumps (neMESYS, Cetoni Gmbh, Korbussen, Germany), the reference fluid was infused at a constant flow rate of *Q_R_* = *Q_0_*. The test fluid was supplied at periodic on-off fashion (period (*T*) = 240 s, duty ratio = 0.5, and *Q_T_* = *Q_0_*). A microfluidic device was placed on an optical inverted microscope (BX51, Olympus, Tokyo, Japan) equipped with a 10× objective lens (NA = 0.25). A high-speed camera (FASTCAM MINI, Photron, Tokyo, Japan) captured two microscopic images sequentially at a frame rate of 500 fps. The image acquisition continued at an interval of 0.5 s with a function generator. All experiments were conducted at a temperature of 25 °C.

### 2.3. Quantification of Interface, Velocity Fields, and Image Intensity

The right side panel in Figure 1A-c shows a microscopic image taken for estimating the interface in a co-flowing channel. A specific region-of-interest (ROI, 240 × 200 pixels) was selected within a straight region of the co-flowing channel. Using MATLAB 2019 (MathWorks, Natick, MA, USA), the blood-filled width (*W_B_*) over the ROI was estimated with Otsu’s method [37]. The interface in the co-flowing channel (*α*) was defined as *α* = *W_B_*/*W*. As shown in Figure 1A-d, the velocity fields of the blood sample in the test fluid channel were obtained with a micro-particle image velocimetry (PIV) technique [38]. A specific ROI (240 × 200 pixels) was selected within the test fluid channel. The size of the interrogation window was 32 × 32 pixels. The window overlap was 50%. The velocity fields within ROI were validated with a local median filter. The average velocity (*<U>_μPIV_*) was obtained by averaging the velocity fields over the ROI. To evaluate the RBC aggregation of the blood sample, the microscopic image intensity of the blood sample flowing in the test fluid channel was quantified with digital image processing. As shown on the right side channel, a specific ROI (240 × 200 pixels) was selected within the test fluid channel. An average image intensity (*<I>*) was obtained by averaging the image intensities distributed over the ROI.

As a preliminary demonstration, three properties of blood sample (i.e., blood viscosity, RBC aggregation, and viscoelasticity) were quantified by analyzing interface in coflowing channel (*α*) and image intensity of blood flows in test channel (*<I>*), respectively. A blood sample (normal RBCs suspended in 1× PBS, Hct = 50%) and glycerin (20%) as the test fluid were prepared to show temporal variations of the interface (*α*) depending on the air cavity in a syringe. Two syringes were filled with the test fluid (~0.5 mL) and reference fluid (~0.5 mL), respectively. Then, the air cavity in the reference fluid syringe was set to 0.1 mL. The air cavity in the test fluid syringe was set to zero. Furthermore, 1× PBS as reference fluid was injected at a constant flow rate of *Q_0_* =1 mL/h. The test fluid was injected in a periodic on-off fashion (*T* = 240 s, duty ratio = 0.5, and *Q_0_* =1 mL/h). Figure 1B-a,b show microscopic images of two test fluids (i.e., blood sample and glycerin (20%)) at specific time instants (*t* = 90, 130, 140, 150, and 230 s). When capturing microscopic images as shown in Figure 1B-b, light intensity increases significantly in order to clearly see RBCs flowing in the test channel. After turning off the syringe pump of the test fluid at *t* =120 s, the blood-filled width (*W_B_*) tended to decrease over time. Figure 1B-c shows temporal variations of the obtained α with respect to blood sample and glycerin (20%), respectively. Under the constant blood flow condition (i.e., *t* < 120 s), the value of *α* of the blood sample was higher than that of glycerin (20%). Under the transient flow condition (i.e., 120 s < t < 240 s), the value of *α* of the blood sample took longer to reach a constant value compared with glycerin (20%). After turning on the syringe pump for the blood sample, the blood flow rate and interface (*α*) remained constant after an elapse of time constant. Blood viscosity was then quantified with the information of the interface. After an elapse of a half period, the syringe pump for the blood sample was set to turn off. The interface decreased substantially over time. After an elapse of the time constant, RBC aggregation occurred and contributed to increasing image intensity of the blood sample. RBC aggregation was then obtained by analyzing temporal variations of image intensity. In other words, blood viscosity and RBC aggregation were obtained at a constant flow rate and extremely low flow rate, respectively. Thus, to measure both properties effectively, it was necessary to secure sufficient duration of constant flow rate and stationary flow rate under periodic on–off operation of the syringe pump. In other words, time constant should remain much smaller than half period. Air compliance was used widely to eliminate fluidic instability resulting from the syringe pump. However, the air compliance tended to increase time constant substantially. When turning on syringe pump (i.e., 0 < *t* < 0.5 *T*), the air cavity (~0.1 mL) inside syringe did not arrive at a constant value of *β*. When turning off the syringe pump (i.e., 0.5 *T* < *t* < *T*), *β* decreased gradually over time. The longer time constant made it difficult to quantify both properties of blood samples. As air compliance hindered in quantifying blood viscosity and RBC aggregation, it is necessary to minimize the time constant by removing the air cavity in the blood syringe pump and securing the air cavity in reference syringe. Thus, the dynamic behavior of two fluids (i.e., time constant) should be considered as a significant factor for effectively quantifying blood viscosity and RBC aggregation under periodic on–off blood flow condition.

## 3. Results and Discussion

### 3.1. Variations of Time Constant with Respect to Air Cavity in Reference Fluid Syringe

According to previous studies [22,23,26,29], air compliance was widely used to stabilize fluidic instability resulting from syringe pumps. In this study, a reference fluid was injected at a constant flow rate with a syringe pump. However, the air cavity in the reference fluid syringe might have an influence on the dynamic variation of the interface in co-flowing channels. For this reason, it was necessary to evaluate the contributions of the air cavity in the reference fluid syringe to time constants of the interface in co-flowing channels. The air cavity in the reference fluid syringe was set to *V_air, R_* = 0, 0.1, and 0.2 mL. To separate the effect of the air cavity in the test fluid syringe, such a cavity was set to zero (*V_air, T_* = 0). Blood samples (normal RBCs suspended in 1× PBS, Hct = 50%) and glycerin (20%) were prepared as test fluids.

**Figure 2:**
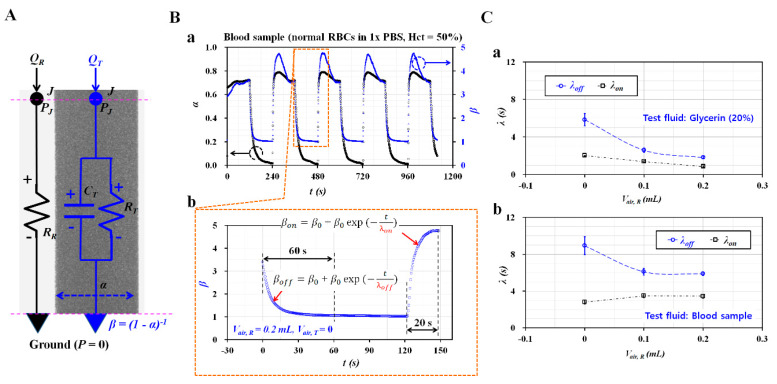
Quantitative evaluations of time constant with respect to the air cavity in the reference fluid syringe (*V_air, R_* = 0, 0.1, and 0.2 mL). Here, the air cavity in the test fluid syringe was set to zero. (**A**) Fluidic circuit model for two fluids (reference fluid, test fluid) flowing in a co-flowing channel. (**B**) Quantifications of time constants (*λ_off_*, *λ_on_*) during each period. (a) Temporal variations of *α* and *β* with respect to *V_air, R_* = 0.2 mL. (b) Quantifications of *λ_off_* and *λ_on_* during turn-on and turn-off operation of the syringe pump. (**C**) Variations of *λ_off_* and *λ_on_* with respect to the test fluids (blood sample and glycerin (20%)) and air cavity in the reference syringe (*V_air, R_* = 0, 0.1 and 0.2 mL). (a) Variations of *λ_off_* and *λ_on_* with respect to *V_air, R_* and glycerin (20%). (b) Variations of *λ_off_* and *λ_on_* with respect to *V_air, R_* and blood sample.

To model the contribution of the air cavity in the syringe to interface, it was required to derive a governing equation for two fluids flowing in a co-flowing channel. As shown in Figure 2A, a fluidic circuit model for two fluids (reference fluid and test fluid) flowing in a co-flowing channel was constructed with discrete circuit elements (i.e., flow rate elements: *Q_R_*, *Q_T_*, resistance elements: *R_R_*, *R_T_*, and compliance element: *C_T_*). Here, *C_T_* denotes the compliance element that was combined with flexible tubing, a microfluidic channel, and an air cavity in the syringe. Additionally, ground (▼) represents pressure set to zero. To keep the mathematical model simple, the interface in the co-flowing channel was modeled as a virtual wall. Different boundary conditions between the real physical model and the mathematical model were compensated by adding a correction factor (*C_f_*) into the governing equation [39]. Thus, both fluids in the co-flowing channel were modeled independently with discrete circuit elements. The governing equation on interface (*α*) for both fluids flowing in the co-flowing channel is expressed as follows:

(1) $$C_{T}R_{WT}\frac{d}{dt}\left(\frac{C_{f}}{1-\alpha }\right)+\frac{C_{f}\;\alpha }{1-\alpha }=\left(\frac{Q_{T}}{Q_{R}}\right)\left(\frac{\mu_{T}}{\mu_{R}}\right)$$

**Figure A1:**
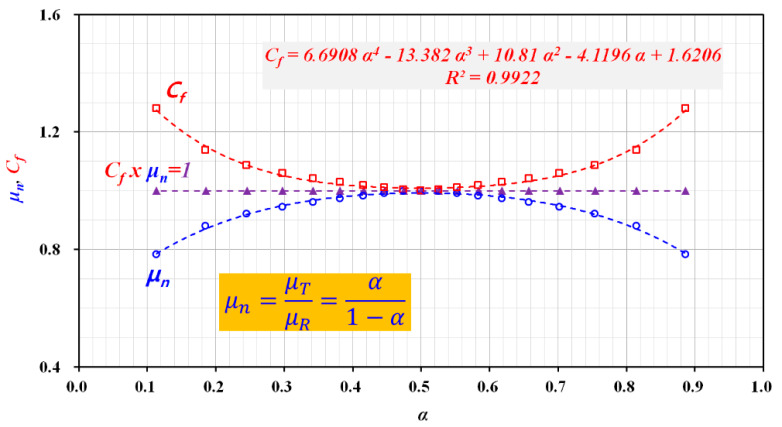
Variations of normalized viscosity (*μ_n_*) with respect to interface (*α*) in coflowing channel. Based on the principle of parallel streams [47], *μ_n_* was expressed as *μ_n_* = *μ_T_*/*μ_R_* = α/(1−α). *μ_T_* and *μ_R_* represent viscosity of test fluid and reference fluid, respectively. As the viscosity of test fluid is assumed as constant with respect to α, correction factor (*C_f_*) was then obtained as *C_f_* = 6.6908 *α^4^* – 13.382 *α^3^* + 10.81 *α^2^* – 4.1196 *α* + 1.6206 (*R^2^* = 0.9922) by using the following relationship (i.e., *C_f_* x *μ_n_* = 1).

Here, $R_{WT}$ is given by $R_{WT}=12\mu_{T}L_{cc}/\left(W_{T}h^{3}\right)$, where $L_{cc}$ denotes the channel length of the co-flowing channel. Subscript T means test fluid. Instead of subscript T, subscript B is also used for representing blood sample. According to a numerical simulation using CFD-ACE+ (Ver. 2019, ESI Group, Paris, France), the correction factor could be approximately expressed as *C_f_* = 6.6908 *α^4^* – 13.382 *α^3^* + 10.81 α^2^ – 4.1196 α + 1.6206 (*R^2^* = 0.9922, 0.1 < *α* < 0.9) (Figure A1, Appendix A). Because of the nonlinear terms in the left member of Equation (1), the differential equation was difficult to solve substantially. Based on an approximate procedure [39], two approximate coefficients (*F_1_*, *F_2_*) were obtained as *F_1_* = 1.112, *F_2_* = 1.129, respectively. Consequently, 1/(1-α) was converted into *β* and Equation (1) was transformed into a linear differential equation as follows:

(2) $$\lambda \frac{d}{dt}\left(\beta \right)+\beta =1+\frac{1}{F_{2}}\left(\frac{Q_{T}}{Q_{R}}\right)\left(\frac{\mu_{T}}{\mu_{R}}\right)$$

In Equation (2), the time constant (*λ*) is expressed as $\lambda =\frac{F_{1}}{F_{2}}C_{T}R_{WT}≅C_{T}R_{WT}$. The compliance element (*C_T_*) presents a linear relation with the time constant (*λ*) and includes the effect of the air cavity in the syringe. Thus, the contribution of the air cavity could be obtained quantitatively by measuring the time constant (*λ*) with transient behaviors of *β*.

As shown in Figure 2B-a, temporal variations of *α* and *β* were obtained with respect to the blood sample (normal RBCs suspended in 1× PBS, Hct = 50%). Here, the air cavity in the reference fluid syringe was set to 0.2 mL (*V_air, R_* = 0.2 mL). Based on Equation (2), the temporal variations of *β* were represented as shown in Figure 2B-b. When sequentially turning syringe pumps on and off, two time constants (*λ_off_*, *λ_on_*) could be obtained by analyzing transient variations of *β.* First, under the turn-off operation of a syringe pump, temporal variations of *β_off_* were extracted for 60 s. Based on an exponential model (i.e., *β_off_* = *β_0_* + *β_1_* exp (-*t*/*λ_off_*)), *λ_off_* was obtained by conducting nonlinear regression analysis with Matlab 2019. Second, under the turn-on operation of a syringe pump, *β_on_* converged in a shorter time interval than for *β_off_*. Temporal variations of *β_on_* were extracted for 20 s. Similarly, based on an exponential model (i.e., *β_on_* = *β_0_* + *β_1_* exp (-*t*/*λ_on_*)), *λ_on_* was obtained by conducting non-linear regression analysis. Figure 2C-a shows variations of *λ_off_* and *λ_on_* with respect to *V_air, R_* and glycerin (20%). All experimental data were expressed as mean ± standard deviation. The error bar represented single standard deviation. Note that *λ_off_* was much longer than *λ_on_* within 0.2 mL of the air cavity. Additionally, *λ_off_* decreased substantially when the cavity volume increased from 0 to 0.1 mL. Above *V_air, R_* = 0.1 mL, it decreased slightly. Figure 2C-b shows variations of *λ_off_* and *λ_on_* with respect to *V_air, R_* and the blood sample. Similar to the glycerin solution, *λ_off_* decreased considerably when the air cavity increased from 0 to 0.1 mL. The air cavity in the reference fluid syringe (~0.1 mL) contributed to decreasing the time constant (*λ_off_*) significantly. However, *λ_on_* did not present distinctive variations with respect to the air cavity in the reference fluid syringe. Additionally, two time constants remained unchanged above *V_air, R_* = 0.1 mL. According to discrete fluidic circuit analysis, air compliance (*C*) plays a role in regulating the alternating component of the flow rate. In this study, flow rate of the reference fluid remained unchanged over time. It was modeled as direct component of flow rate. Thus, air cavity secured in reference syringe did not contribute to the changing time constant. However, air cavity with 0.1 mL decreased time constant substantially. Taking into account the fact that air compliance caused the time constant to increase generally, the result showed different trends. Above a 0.1 mL air cavity, the time constant varied slightly. The constant value of the time constant was obtained through fluid viscosity and the compliance effect of the tubing and PDMS device. According to these experimental results, the air cavity in the reference fluid syringe (~0.1 mL) contributed to decreasing *λ_off_* greatly. Note that *λ_off_* decreased more significantly than *λ_on_*. Above an air cavity volume of 0.1 mL, the time constants did not present substantial variation.

### 3.2. Valuations of Time Constant with Respect to Hematocrit and Air Cavity in Blood Sample Syringe

**Figure 3:**
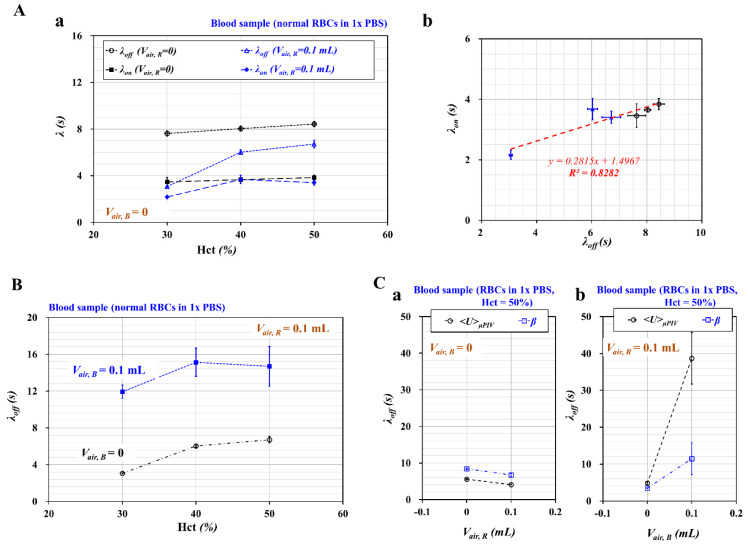
Quantitative evaluations of time constants (*λ_off_*, *λ_on_*) with respect to hematocrit and air cavity in each syringe. Here, a blood sample was prepared by adding normal RBCs into 1× PBS. (**A**) The comparison of two time constants with respect to hematocrit and air cavity. (a) Variations of *λ_off_* and *λ_on_* with respect to Hct = 30%, 40%, and 50% and *V_air, R_* = 0 and 0.1 mL. (b) Linear relationship between *λ_off_* and *λ_on_*. Here, the air cavity in the blood sample syringe was set to zero. (**B**) Variations of *λ_off_* and *λ_on_* with respect to Hct = 30%, 40%, and 50% with *V_air, B_* = 0 and 0.1 mL. Here, the air cavity in the reference fluid syringe was set to 0.1 mL. (**C**) Quantitative comparison of *λ_off_* obtained from *<U>_μPIV_* and *β*. The hematocrit of the blood sample was adjusted to Hct = 50% by adding normal RBCs into 1× PBS. (a) Comparison of *λ_off_* obtained from *<U>_μPIV_* and *β* with respect to *V_air, R_* = 0 and 0.1 mL. Here, the air cavity in the blood sample syringe was set to zero. (b) Comparison of *λ_off_* obtained from *<U>_μPIV_* and *β* with respect to *V_air, B_* = 0 and 0.1 mL. Here, the air cavity in the reference fluid syringe was set to 0.1 mL.

First, to evaluate the contribution of hematocrit to the time constant, the blood sample (Hct = 30%, 40%, and 50%) was prepared by adding normal RBCs into 1× PBS. As shown in Figure 3A-a, variations of *λ_off_* and *λ_on_* were obtained with respect to Hct. To evaluate the contribution of the air cavity in the reference fluid syringe, such a cavity was set to *V_air, R_* = 0 and 0.1 mL. The air cavity in the blood sample syringe was set to *V_air, B_* = 0. In contrast with *λ_on_*, *λ_off_* increased largely with respect to Hct. In addition, the air cavity in the reference fluid syringe contributed to decreasing the time constant substantially. As shown in Figure 3A-b, a scatter plot was constructed by plotting *λ_on_* on *Y*-axis and *λ_off_* on *X*-axis. According to linear regression analysis, the following linear regression formula was obtained: *λ_on_ =* 0.2815 *λ_off_* + 1.4967 (*R^2^* = 0.8282). The high regression coefficient (*R^2^*) denotes that *λ_on_* and *λ_off_* showed a strong linear relationship. From these results, *λ_off_* was selected as the representative time constant throughout this study.

Second, to evaluate the effect of the air cavity in the blood sample syringe (*V_air, B_*) on the time constant (*λ_off_*), such cavity was set to *V_air, B_* = 0, and 0.1 mL. Additionally, to stabilize the fluidic instability resulting from the syringe pump, the air cavity in the reference fluid syringe was set to *V_air, R_* = 0.1 mL. As shown in Figure 3B, variations of *λ_off_* were obtained with respect to Hct = 30%, 40%, and 50% and *V_air, B_* = 0, and 0.1 mL. The air cavity in the blood sample syringe contributed to increasing the time constant substantially. Theoretically, the size of syringe pump did not contribute to the varying time constant. According to the previous study [22], the time constant tended to increase linearly with respect to air cavity volume. In other words, air cavity secured in each syringe varied dynamic behaviors of *β* in coflowing channels (i.e., time constant). Thus, it is necessary to fix air cavity secured in each syringe. Note that, interestingly, the air cavity in the reference fluid syringe contributed to decreasing *λ_off_*, as shown in Figure 3A-a. From these results, we inferred that the air cavity increased or decreased the time constant depending on whether it existed in the reference fluid syringe or the blood sample syringe.

Third, to compare the time constant with temporal variations of *β*, the time constant was additionally obtained with temporal variations of the average velocity of the blood flow in the test fluid channel (*<U>_μPIV_*). A blood sample (Hct = 50%) was prepared as the test fluid by adding normal RBCs into 1× PBS. Figure 3C-a shows *λ_off_* of *<U>_μPIV_* and *λ_off_* of *β* with respect to *V_air, R_* = 0 and 0.1 mL. Here, the air cavity in the blood sample syringe was set to zero. Consequently, *λ_off_* tended to decrease with respect to *V_air, R_*. Both *<U>_μPIV_* and *β* exhibited a similar trend of *λ_off_* with respect to *V_air, R_.* Figure 3C-b shows a comparison of *λ_off_* obtained from *<U>_μPIV_* and *β* with respect to *V_air, B_* = 0 and 0.1 mL. Here, the air cavity in the reference fluid syringe was set to 0.1 mL. Consequently, *λ_off_* tended to increase with respect to *V_air, B_*. Both *<U>_μPIV_* and *β* exhibited increase in *λ_off_* significantly with respect to *V_air_,_B_*. The time constant obtained with *β* presented a very similar trend with respect to the air cavity compared with the time constant obtained with *<U>_μPIV_*. As quantification of *<U>_μPIV_* required an expensive high-speed camera and much time for the micro-PIV procedure, the quantification of *β* could be considered more effective.

Finally, to evaluate the contribution of the air cavity in the blood sample syringe to blood viscosity (*μ_B_*), the value of *μ_B_* was obtained with respect to *V_air, B_* = 0 and 0.1 mL. The blood viscosity was quantified under constant flow rate; both fluids were infused at the same flow rate (i.e., *Q_B_* = *Q_R_*). By setting $\frac{d\beta }{dt}=0$ in Equation (2), a formula of blood viscosity was derived as follows:

(3) $$\mu_{B}=\mu_{R}\times \left(\beta -1\right)\times F_{2}$$

**Figure 4:**
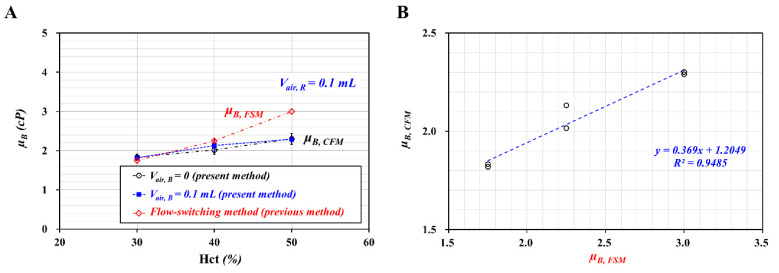
Quantitative comparison of blood viscosity (*μ_B_*) between the co-flowing method (present method) and a flow switching method (previous method). To evaluate the effect of *V_air, B_* on *μ_B_*, *V_air, B_* was varied from 0 to 0.1 mL. Here, *V_air, R_* was set to 0.1 mL. (**A**) Variations of *μ_B_* obtained with two different methods with respect to Hct. (**B**) The inset shows the linear relationship between *μ_B, CFM_* (co-flowing method) and *μ_B, FSM_* (flow switching method).

As shown in Figure 4A, *μ_B_* tended to increase with respect to Hct. As expected, the air cavity in the blood sample syringe did not contribute to varying blood viscosity. In addition, it was inferred that the air cavity in the reference fluid syringe (~0.1 mL) was sufficient to maintain a constant flow rate, even at *V_air, B_* = 0.

To compare with the blood viscosity obtained with the present method (i.e., co-flowing method), the blood viscosity of the same blood sample was also obtained with a previous method (i.e., flow-switching method) [40]. The previous method produced a higher value of blood viscosity than the present method. The Fåhræus–Lindqvist effect indicated that blood viscosity varied with respect to channel diameter. In other words, blood viscosity tended to decrease at a smaller channel due to the existence of a cell-free layer. However, blood viscosity remained constant for wider channel with above 300~500 μm. Here, the contribution of a cell-free layer was negligible because it was much smaller than the channel size. As a rectangular channel (width = *W*, and depth = *h*) was filled with a blood sample, an equivalent circular diameter (*d*) was estimated as $d=\sqrt{\frac{4\;W\cdot h}{\pi }}$ with mass conservation. For the previous method (i.e., switching flow method), a single fluidic channel was filled with blood sample completely when reversal flow in junction occurred. Then, equivalent diameter was estimated as *d* = 358 μm. However, for the present method (i.e., co-flowing method), the corresponding interface of each hematocrit was obtained as *α* = 0.65 ± 0.01 for Hct = 30%, α = 0.67 ± 0.01 for Hct = 40%, and α = 0.68 ± 0.01 for Hct = 50%. The equivalent diameter was then estimated as *d* = 288~294 μm. According to the previous study [41], for channel diameter with below *d* = 400 μm, blood viscosity tended to decrease gradually with respect to equivalent diameter. Because the present method had smaller equivalent diameter than the previous method, it was reasonable that blood viscosity obtained by the present method was underestimated substantially when compared with blood viscosity obtained by the previous method. To obtain a linear relationship between both methods, as shown in the inset of Figure 4B, a scatter plot was constructed by plotting the viscosity obtained by the present method (i.e., co-flowing method with *μ_B, CFM_*) on *Y*-axis and the viscosity obtained by previous method (i.e., flow-switching method: *μ_B, FSM_*) on *X*-axis. According to regression analysis, a linear regression formula was obtained: *μ_B, CFM_* = 0.369 *μ_B, FSM_* + 1.2049 (*R^2^* = 0.9485). The high value of the regression coefficient (*R^2^*) means that the co-flowing method (i.e., the present method) could be used effectively to monitor blood viscosity compared with the flow-switching method (i.e., the previous method).

### 3.3. Quantitative Evaluations of Image Intensity, Blood Velocity, and Interface with Respect to Diluent

To evaluate variations of mechanical properties of a blood sample at constant blood flow rate, a blood sample (Hct = 50%) was prepared by adding normal RBCs into two different diluents, namely 1× PBS and dextran solution (10 mg/mL). Here, the dextran solution was used as a diluent to enhance the RBC aggregation in the blood sample. The contribution of the dextran solution to the mechanical properties of the blood sample was evaluated by measuring image intensity (*<I>*), average velocity (*<U>_μPIV_*), and interface (*α* =1 - *β^−1^*) with respect to the blood flow rate (or shear rate). Using two syringe pumps, both fluids were injected at the same flow rate (*Q_R_* = *Q_B_* = *Q_sp_*). The air cavity in each syringe was set to 0.1 mL (i.e., *V_air, R_* = *V_air, B_* = 0.1 mL).

**Figure 5:**
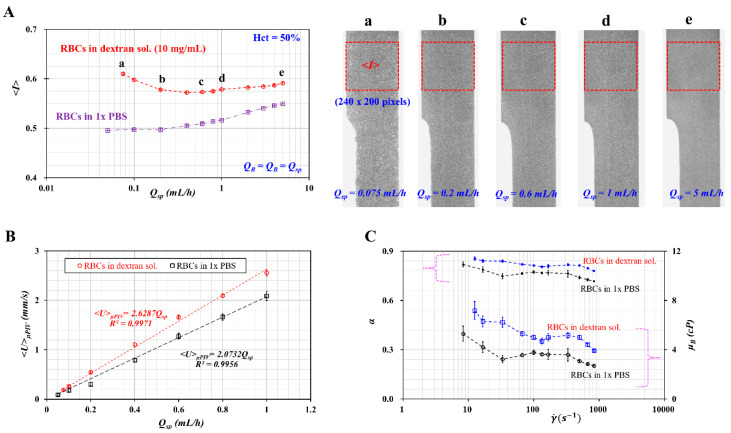
Quantitative evaluations of image intensity (*<I>*), blood velocity (*<U>_μPIV_*), and interface (*α*) with respect to diluent. Blood samples (Hct = 50%) were prepared by adding normal RBCs into different diluents, namely 1× PBS and dextran solution (10 mg/mL). The flow rate of each fluid was fixed at the same flow rate (*Q_R_* = *Q_B_* = Q*_sp_*). (**A**) Variations of microscopic image intensity (*<I>*) with respect to *Q_sp_* and diluent. The value of *<I>* was obtained by averaging the image intensity distributed over a specific region-of-interest (ROI) (240 × 200 pixels) selected within the test fluid channel. The right side panel shows microscopic images captured at a specific flow rate ((a) *Q_sp_* = 0.075 mL/h, (b) *Q_sp_* = 0.2 mL/h, (c) *Q_sp_* = 0.6 mL/h, (d) *Q_sp_* = 1 mL/h, and (e) *Q_sp_* = 5 mL/h). (**B**) Variations of *<U>_μPIV_* with respect to *Q_sp_* and diluent. (**C**) Variations of *α* and *μ_B_* with respect to $\dot{\gamma }$ and diluent.

As shown in Figure 5A, the variation of image intensity (*<I>*) was obtained with respect to *Q_sp_* and the diluent. The right side panel in the figure shows microscopic images captured at specific flow rates (*Q_sp_*): (a) *Q_sp_* = 0.075 mL/h, (b) *Q_sp_* = 0.2 mL/h, (c) *Q_sp_* = 0.6 mL/h, (d) *Q_sp_* = 1 mL/h, and (e) *Q_sp_* = 5 mL/h. For the dextran solution as diluent, *<I>* decreased gradually up to *Q_sp_* = 0.4 mL/h. RBC aggregation caused to increase *<I>* at a lower flow rate. However, when the flow rate increased, RBCs tended to disaggregate. Above *Q_sp_* = 0.6 mL/, *<I>* tended to increase gradually with respect to *Q_sp_*. According to a previous study, the orientation and deformability of RBCs contribute to increasing image intensity [42]. Given that RBCs in 1× PBS did not include RBC aggregation, *<I>* did not increase, even at lower flow rates. The value of *<I>* tended to increase gradually by increasing the flow rate.

**Figure A2:**
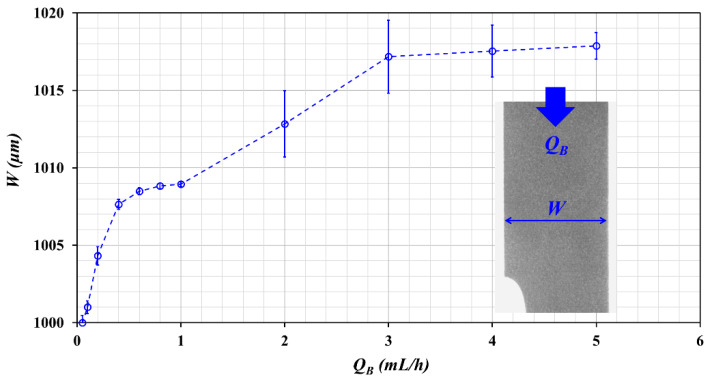
Variations of channel width under blood flow rate (*Q_B_*).

While measuring blood viscosity accurately, it is necessary to evaluate the effect of flow rate on interface (*α* = *W_B_*_/_*W*) in the coflowing channel. As shown in Figure 1A-c, blood-filled width (*W_B_*) could be obtained accurately by conducting image processing. However, the channel width was assumed as *W* = 1000 μm. Maximum flow rate was estimated as 2 mL/h when test fluid and reference fluid were set to the same flow rate of 1 mL/h. While infusing the blood sample into single microfluidic channel, the deformed channel width was quantified by increasing flow rate. Variation of *W* was obtained by varying flow rate (*Q_B_* = 0.05, 0.1, 0.2, 0.4, 0.6, 0.8, 1, 2, 3, 4, and 5 mL/h). As shown in Figure A2 (Appendix A), the channel width of the corresponding flow rate was quantified as *W* = 1009 ± 0.2 μm (*Q_B_* = 1 mL/h), *W* = 1012.8 ± 2.1 μm (*Q_B_* = 2 mL/h), and *W* = 1017.5 ± 1.7 μm (*Q_B_* = 4 mL/h). From the results, variation of channel width was estimated as less than 2% under the maximum flow rate of 2 mL/h.

Variations of *<U>_μPIV_* with respect to *Q_sp_* and diluent were obtained, as shown in Figure 5B. The value of *<U>_μPIV_* tended to increase linearly with respect to *Q_sp_*. According to linear regression analysis, a linear regression formula for each diluent was obtained: *<U>_μPIV_* = 2.6287 *Q_sp_* (*R^2^* = 0.9971) for dextran solution (10 mg/mL) and *<U>_μPIV_* = 2.0732 *Q_sp_* (*R^2^* = 0.9956) for 1× PBS. These results indicated that RBCs suspended in dextran solution reached a higher value of *<U>_μPIV_* (~26.8%) compared with RBCs suspended in 1× PBS.

Finally, to evaluate variations of interface (α) with flow rate and diluent, variations of *α* and *μ_B_* were obtained with respect to shear rate and diluent. For a rectangular channel (width = *W*, and depth = *h*) with low aspect ratio [8], a shear rate for each flow rate (*Q_sp_*) is given approximately by $\dot{\gamma }=\frac{6Q_{sp}}{W\;h^{2}}$. Using Equation (3), the blood viscosity of the blood sample was obtained in terms of the shear rate. As shown in Figure 5C, the interface (*α*) of RBCs suspended in the dextran solution reached a higher value of interface compared with RBCs suspended in 1× PBS. The blood viscosity decreased gradually with respect to the shear rate. The blood sample behaved as a non-Newtonian fluid (or shear-thinning fluid). Furthermore, a dextran solution (10 mg/mL) as diluent contributed to increasing the blood viscosity significantly compared with 1× PBS. When compared with previous results [8], our results showed consistent trends with respect to diluents.

### 3.4. Variations of Red Blood Cells (RBC) Aggregation, Viscosity, and Viscoelasticity with Respect to Diluent and Air Cavity in Syringe

To quantify three mechanical properties of blood sample (RBC aggregation, viscosity, and viscoelasticity) with respect to air cavity (or air compliance), variations of *<I>*, *<U>_μPIV_*, and *β* were simultaneously obtained with respect to diluent and air cavity in each syringe. A blood sample (Hct = 50%) was prepared by adding normal RBCs into four different diluents, namely 1× PBS, two dextran solutions (5, and 10 mg/mL), and plasma. The air cavity in the reference fluid syringe was fixed at *V_air, R_* = 0.1 mL. Additionally, the air cavity in the blood sample syringe varied from *V_air, B_* = 0 to *V_air, B_* = 0.1 mL. Based on experimental results shown in Figure 5A, the flow rate of each fluid was reset to *Q_0_* = 0.5 mL/h for measuring RBC aggregation effectively.

**Figure 6:**
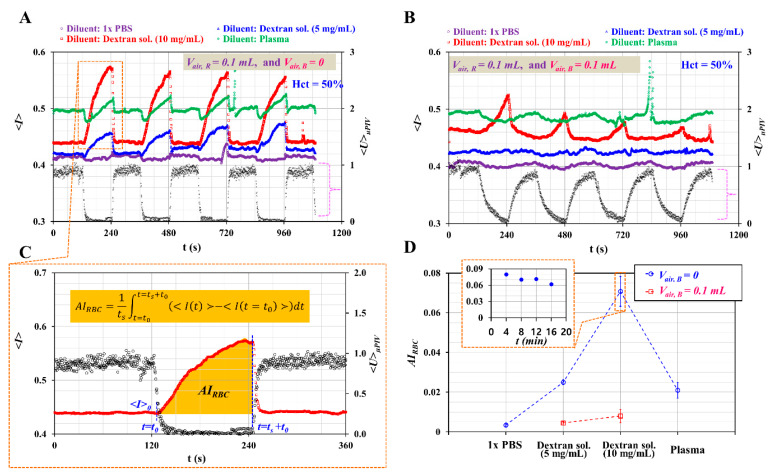
Quantitative evaluations of RBC aggregation with respect to diluent and air cavity in the blood sample syringe. Here, the flow rate of the syringe pump was reset to *Q_0_* = 0.5 mL/h. The air cavity in the reference fluid syringe was set to 0.1 mL. (**A**) Temporal variations of *<I>* and *<U>_μPIV_* with respect to diluent, i.e., 1× PBS, two dextran solution (5, and 10 mg/mL), and plasma. The air cavity in the blood sample syringe was set to zero. (**B**) Temporal variations of *<I>* and *<U>_μPIV_* with respect to diluent. The air cavity in the blood sample was set to 0.1 mL. (**C**) Quantification of RBC aggregation index (*AI_RBC_*). The value of *AI_RBC_* was obtained as $AI_{RBC}=\frac{1}{t_{s}}\int_{t=t_{0}}^{t=t_{0}+t_{s}}\left(<I\left(t\right)>-<I\left(t=t_{0}\right)>\right)dt$. (**D**) Variations of *AI_RBC_* with respect to diluent and *V_air, B_*. The inset shows temporal variations of *AI_RBC_* with respect to dextran solution (10 mg/mL).

First, as shown in Figure 6A, temporal variations of *<I>* and *<U>_μPIV_* were obtained with respect to diluents. Here, the air cavity in the blood sample syringe was set to *V_air, R_* = 0. When the syringe pump was turned off periodically, *<U>_μPIV_* decreased suddenly over time. RBC aggregation increased *<I>* gradually over time. Given that 1× PBS did not stimulate RBC aggregation, *<I>* of 1× PBS remain unchanged over time. However, two dextran solutions contributed to increasing *<I>* over time substantially. Given that plasma proteins contributed to RBC aggregation [43,44], *<I>* of plasma increased gradually over time.

To evaluate the effect of air compliance on RBC aggregation, the air cavity in the blood sample syringe was varied from *V_air, B_* = 0 to *V_air, B_* = 0.1 mL. As shown in Figure 6B, temporal variations of *<I>* and *<U>_μPIV_* were obtained with respect to diluent. Even when turning off the syringe pump, *<U>_μPIV_* tended to decrease gradually over time. For this reason, except for a higher concentration of dextran solution (10 mg/mL), *<I>* did not show substantial increase over time. From these results, the air cavity (~0.1 mL) in the blood sample syringe delayed the transient behaviors of blood velocity considerably. Thus, it was inferred that air compliance hindered the quantification of RBC aggregation. To quantify RBC aggregation with *<I>*, it was necessary to define an RBC aggregation index. From Figure 6A, temporal variations of *<I>* and *<U>_μPIV_* were redrawn from *t* = 0 to *t* = 360 s. As shown in Figure 6C, after *t* = 120 s (i.e., turn-off operation of the syringe pump), *<U>_μPIV_* decreased largely over time. Note also that *<I>* tended to increase gradually over time. Here, a specific time instant and minimum value of *<I>* were denoted as *t* = *t_0_* and < *I (t = t_0_)* >, respectively. The RBC aggregation index was then obtained by analyzing *<I>* from *t* = *t_0_* to *t* = *t_0_* + *t_s_*. According to a previous study [45], an RBC aggregation index (*AI_RBC_*) can be defined as follows:

(4) $$AI_{RBC}=\frac{1}{t_{s}}\int_{t=t_{0}}^{t=t_{0}+t_{s}}\left(<I\left(t\right)>-<I\left(t=t_{0}\right)>\right)dt$$

Based on the temporal variations of *<I>* shown in Figure 6A,B, *AI_RBC_* was quantified at periodic intervals (*T* = 240 s). Figure 6D shows variations of *AI_RBC_* with respect to diluent and *V_air, B_*. The inset of Figure 6D shows temporal variations of *AI_RBC_* with respect to dextran solution (10 mg/mL) and *V_air, B_* = 0. The RBC aggregation index was quantified with repetitive tests (n = 8) and expressed as mean ± standard deviation. Under no air cavity in the blood sample syringe, the RBC aggregation index for each diluent was obtained as *AI_RBC_* = 0.003 ± 0.001 for 1× PBS, *AI_RBC_* = 0.025 ± 0.001 for dextran solution (5 mg/mL), *AI_RBC_* = 0.071 ± 0.008 for dextran solution (10 mg/mL), and *AI_RBC_* = 0.021 ± 0.003 for plasma. The dextran solutions and plasma contributed to increasing the RBC aggregation index significantly compared with 1× PBS. Additionally, *AI_RBC_* tended to increase significantly at higher concentration of dextran solution. When the air cavity in the blood sample syringe was reset to 0.1 mL, the RBC aggregation index for the two dextran solutions was obtained as *AI_RBC_* = 0.004 ± 0.001 for the first dextran solution (5 mg/mL) and *AI_RBC_* = 0.008 ± 0.003 for the second dextran solution (10 mg/mL). Given that the air cavity (or air compliance) tended to delay the transient behavior of the blood velocity, *AI_RBC_* decreased considerably. From these results, the air cavity (~0.1 mL) in the blood sample syringe hindered the quantification of the RBC aggregation substantially.

**Figure 7:**
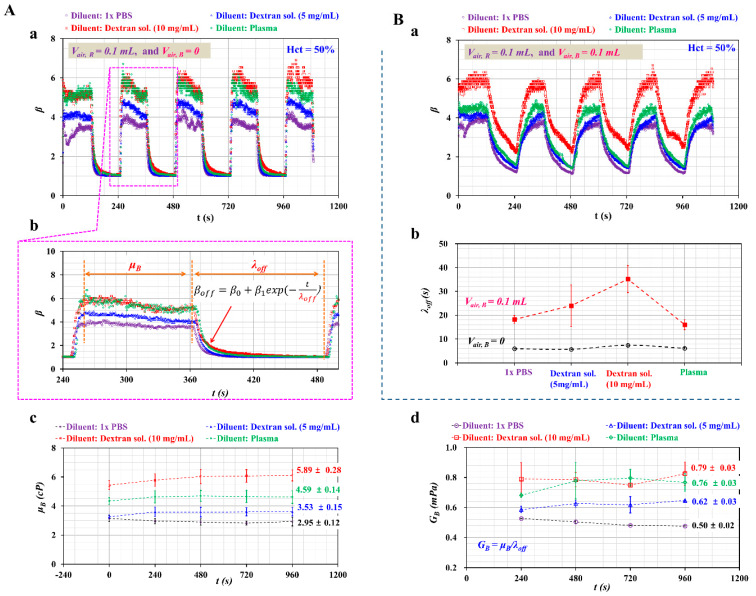
Quantitative evaluations of viscosity (*μ_B_*) and time constant (*λ_off_*) with respect to diluent and air cavity in the blood syringe. Here, the flow rate of the individual syringe pump and the air cavity in the reference fluid syringe were fixed at *Q_0_* = 0.5 mL/h and *V_air, R_* = 0.1 mL, respectively. (**A**) Variations of viscosity (*μ_B_*) and elasticity (*G_B_*) with respect to diluent and *V_air, B_* = 0. (a) Temporal variations of *β* with respect to diluent and *V_air, B_* = 0. (b) Quantification of *μ_B_* and *λ_off_* during a single period. Here, *μ_B_* and *λ_off_* were obtained sequentially by turning on and off the syringe pump. (c) Variations of *μ_B_* at intervals of 240 s. (d) Variations of *G_B_* with respect to diluent. Here, *G_B_* was obtained by dividing *μ_B_* by *λ_off_*. (**B**) Variations of *λ_off_* with respect to diluent and *V_air, B_* = 0.1 mL. (a) Temporal variations of *β* with respect to diluent. (b) Variations of *λ_off_* with respect to diluent and *V_air,_* *_B_*.

Second, as shown in Figure 7A-a, the temporal variations of *β* were obtained with respect to diluent. Air cavities of each syringe were set to *V_air, R_* = 0.1 mL and *V_air, B_* = 0, respectively. Among the values of *β* obtained in Figure 7A-a, to represent how the blood viscosity (*μ_B_*) and the time constant (*λ_off_*) were quantified over a single period, temporal variations of *β* were redrawn at specific durations ranging from *t* = 240 s to *t* = 500 s.

As shown in Figure 7A-b, the value of *μ_B_* was obtained with the Equation (3) under turn-on operation of the syringe pump (*t* < 0.5 *T*). Afterward, *λ_off_* was estimated with regression analysis (*β_off_* = *β_0_* + *β_1_* exp (-*t*/*λ_off_*) under turn-off operation of the syringe pump (0.5 *T* < *t* < *T*). As shown in Figure 7A-c, variations of *μ_B_* were obtained at intervals of 240 s with respect to diluent. The value of *μ_B_* for each diluent was obtained as *μ_B_* = 2.95 ± 0.12 cP for 1× PBS, *μ_B_* = 3.53 ± 0.15 cP for the first dextran solution (5 mg/mL), *μ_B_* = 5.89 ± 0.28 cP for the second dextran solution (10 mg/mL), and *μ_B_* = 4.59 ± 0.14 cP for plasma. Under the turn-off operation of the syringe pump, the time constant (*λ_off_*) was obtained with respect to diluent. Using a linear Maxwell model (i.e., *λ_off_* = *μ_B_*/*G_B_*), *G_B_* was obtained by dividing *μ_B_* by *λ_off_*. As shown in Figure 7B-b, variations of *λ_off_* were represented with respect to diluent and *V_air, B_* = 0. Additionally, Figure 7A-d shows variations of elasticity (*G_B_*) with respect to diluent. The value of *G_B_* for each diluent was obtained as *G_B_* = 0.5 ± 0.02 mPa for 1× PBS, *G_B_* = 0.62 ± 0.03 mPa for the first dextran solution (5 mg/mL), *G_B_* = 0.79 ± 0.03 mPa for the second dextran solution (10 mg/mL), and *G_B_* = 0.76 ± 0.05 mPa for plasma. From these results, blood viscoelasticity (viscosity, elasticity) was quantified consistently with respect to diluent under the air cavity in each fluid syringe (*V_air, R_* = 0.1 mL, and *V_air, B_* = 0). The dextran solution as diluent contributed to increasing viscosity and elasticity substantially compared with 1× PBS. The plasma reached a higher value of viscosity and elasticity compared with 1× PBS. In other words, the plasma proteins led to increased viscosity and elasticity.

To quantify the effect of the air cavity in the blood sample syringe on *β*, the volume of such air cavities was varied from 0 to 0.1 mL. Additionally, the volume of the air cavity in the reference fluid syringe was set to 0.1 mL. As shown in Figure 7B-a, temporal variations of *β* were obtained with respect to diluent. The air cavity in the blood sample syringe delayed the transient behavior of *β* substantially. During turn-on and turn-off operation of the syringe pump, *β* did not reach a constant value within a specific duration. Given that Equation (3) as blood viscosity was effective for blood viscosity only for constant values of *β*, it was impossible to obtain the blood viscosity with no information on flow rate (or velocity) at a specific time instant. Figure 7B-b shows variations of *λ_off_* with respect to diluent and *V_air, B_*. Note that the case *V_air, B_* = 0.1 mL contributed to increasing *λ_off_* significantly compared with *V_air, B_* = 0. When setting *V_air, B_* = 0.1 mL, *λ_off_* was increased largely at a higher concentration of dextran solution.

As shown in Figure 7B-b, while increasing air cavity was secured in the blood syringe from 0 to 0.1 mL, the corresponding time constant of each diluent increased about *Δλ_off_* = 13.3 s (1× PBS), *Δλ_off_* = 18.4 s (dextran sol. 5 mg/mL), *Δλ_off_* = 27.8 s (dextran sol. 10 mg/mL), and *Δλ_off_* = 10 s (plasma). As transient time increased largely within a half period, *β* did not arrive to constant value under periodic on-off operation of syringe pump. As a solution, it is necessary to increase period of blood flow rate. Taking into account the fact that time constant increased about 10~27.8 s for each diluent, half period of blood flow rate should increase at least 27.8 s. When the period of blood flow rate changes from *T* = 240 s to *T* = 300 s, it will be inferred that *β* exhibits similar trends as shown in Figure 7A-a. Thus, blood viscosity and RBC aggregation will be obtained without additional information on temporal variations of blood velocity.

**Figure 8:**
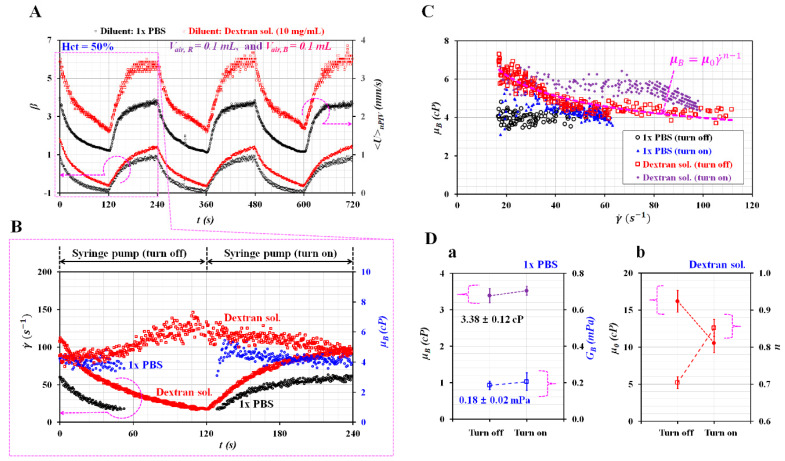
Quantitative evaluation of viscoelasticity with respect to diluent and *V_air, B_* = 0.1 mL/h. A blood sample (Hct = 50%) was prepared by adding normal RBCs into diluent, i.e., 1× PBS and dextran solution (10 mg/mL). The flow rate of each syringe pump was set to *Q_0_* = 0.5 mL/h. The air cavity of the reference fluid syringe was set to 0.1 mL. (**A**) Temporal variations of *β* and *<U>_μPIV_* with respect to diluent. (**B**) Temporal variations of $\dot{\gamma }$ and *μ_B_* with respect to diluent during a single period of 240 s. (**C**) Variations of *μ_B_* with respect to diluent and syringe operation (turn-off and turn-on). (**D**) Variations of viscoelasticity with respect to diluent and pump operation. (a) Variations of $\mu_{B}$ and *G_B_* with respect to 1× PBS and syringe operation. (b) Variations of $\mu_{0}$ and *n* with respect to dextran solution (10 mg/mL) and syringe operation.

To quantify blood viscosity under varying blood flows, it was necessary to obtain *β* and *<U>_μPIV_* over time. Figure 8A shows temporal variations of *β* and *<U>_μPIV_* with respect to two diluents, namely 1× PBS and dextran solution (10 mg/mL). As shown in Figure 5B, the relationship between *<U>_μPIV_* and *Q_sp_* was obtained in advance as a linear regression formula with respect to each diluent, and the average velocity of the blood sample (*<U>_μPIV_*) was converted into the blood flow rate with a regular formula.

Given that the flow rate of the blood sample varied over time, the formula of blood viscosity was corrected as *μ_B_* = *μ_R_* × (*β* − 1) × *F_2_* × (*Q_R_*/*Q_B_*) by adding a flow rate term into Equation (3). Figure 8B shows temporal variations of $\dot{\gamma }$ and *μ_B_* with respect to diluent during a single period of 240 s. Note that *μ_B_* presented large scattering at *Q_sp_* < 0.1 mL/h. Thus, the minimum value of *Q_sp_* was set to 0.1 mL/h. As shown in Figure 8C, variations of *μ_B_* were obtained with respect to $\dot{\gamma }$ during the turn-on and turn-off operation of the syringe pump. For 1× PBS as diluent, *μ_B_* remained constant with respect to the shear rate. However, for the dextran solution as diluent, *μ_B_* decreased gradually with respect to the shear rate. As shown in Figure 8D-a, variations of *μ_B_* and *G_B_* were obtained with respect to the syringe operation (i.e., turn-on, turn-off). Given that *μ_B_* remained unchanged over the shear rate, *G_B_* was calculated by dividing *μ_B_* by the time constant (i.e., *G_B_* = *μ_B_*/*λ_off_* for turn-off operation, and *G_B_* = *μ_B_*/*λ_on_* for turn-on operation). The value of *μ_B_* for each operation was obtained as *μ_B_* =3.38 ± 0.19 cP for the turn-off operation, and *μ_B_* = 3.52 ± 0.12 cP for the turn-on operation. Additionally, the value of *G_B_* for each operation was obtained as *G_B_* = 0.18 ± 0.02 cP for the turn-off operation, and *G_B_* = 0.21 ± 0.05 cP for the turn-on operation. From these results, *μ_B_* and *G_B_* remained unchanged irrespective of the syringe operation. Compared with the results obtained at *V_air, B_* = 0 as showed in Figure 7A-c, a 0.1-mL air cavity (~0.1 mL) in the blood sample syringe caused to overestimate *μ_B_*_._ However, it caused *G_B_* to be underestimated. Figure 8D-b shows variations of $\mu_{0}$ and *n* with respect to the first dextran solution (10 mg/mL) and syringe operation. The constant $\mu_{0}$ decreased significantly by switching the syringe pump from turn-off operation to turn-on operation. In addition, the turn-on operation caused to increase the index *n* substantially compared with the turn-off operation.

In this study, three mechanical properties of blood sample (viscosity, RBC aggregation, and time constant) were quantified with methods suggested in previous studies. First, cone-and-plate viscometer as conventional method has been used to measure blood viscosity. According to the quantitative comparison between conventional viscometer and microfluidic viscometer [40,41,46,47], the previous studies indicated that blood viscosity could be measured consistently in a microfluidic environment. Based on the previous study, as shown in Figure 4A, co-flow method (present method) and flow switching method (previous method) were used to obtain blood viscosity with respect to hematocrit. The present method underestimated blood viscosity when compared with the previous method. However, as shown in Figure 4B, both methods exhibited a high degree of linear relationship (i.e., *R^2^* = 0.9485). Second, RBC aggregation as a conventional method has been quantified by analyzing light intensity [48] or electric impedance [49] of blood sample flowing in slit channel. According to quantitative comparison study [50,51], microscopic image intensity exhibited variations of RBC aggregation in microfluidic channel sufficiently. Thus, without quantitative comparison study, variations of RBC aggregation were quantified by analyzing image intensity of blood flows in the test channel under turn-off blood flows. Finally, under transient flow conditions, time constant has been obtained by analyzing temporal variations of physical parameters including blood velocity, flow rate, and pressure. Based on Equation (2), time constant (*l*) was obtained by analyzing temporal variations of *β*. Furthermore, to compare with time constant obtained from information of *β,* time constant was quantified additionally by analyzing temporal variations of blood velocity (*<U>*). As shown in Figure 3C, both time constants exhibited consistent variations with respect to air cavity in reference syringe.

From these experimental results, it leads to the conclusion that the air cavity in the blood sample syringe made the RBC aggregation and blood viscoelasticity vary substantially. The RBC aggregation index decreased largely, even for a 0.1-mL air cavity in the blood sample syringe because of a longer transient behavior of blood flows. Thus, to measure RBC aggregation and viscoelasticity of blood samples consistently, a 0.1-mL air cavity must be secured in the reference fluid syringe as a minimum condition. Additionally, the air cavity in the blood sample syringe must be minimized as much as possible.

## 4. Conclusions

In this study, the air compliance effect on measurement of blood mechanical properties was quantified experimentally with respect to the air cavity in two driving syringes. Under periodic on–off blood flows, three mechanical properties of blood samples, namely RBC aggregation, blood viscosity, and time constant, were obtained sequentially by quantifying microscopic image intensity of blood samples (*<I>*) flowing in the test channel and the interface (*α*) in a co-flowing channel. Based on a differential equation derived with a fluid circuit model, the time constant was obtained by analyzing temporal variations of *β* = 1/(1–*α*). First, the air cavity in the reference fluid syringe (~0.1 mL) contributed to decreasing *λ_off_* greatly. The *λ_off_* decreased more significantly than *λ_on_*. Above an air cavity volume of 0.1 mL, the time constants did not present substantial variation. The air cavity increased or decreased the time constant depending on whether it existed in the reference fluid syringe or the blood sample syringe. Second, the air cavity did not contribute to varying blood viscosity. From the quantitative comparison study, the co-flowing method (i.e., the present method) could be used effectively to monitor blood viscosity compared with the flow-switching method (i.e., the previous method). Third, given that the air cavity in the blood sample syringe contributed to delaying transient behaviors of blood flows considerably, this hindered the quantification of the RBC aggregation and blood viscosity. As a solution, when the period of blood flow rate increases about twice time constant (i.e., *ΔT* = 2*λ_off_*), blood viscosity and RBC aggregation could be obtained without additional information on temporal variations of blood velocity. From these experimental results, to measure the aforementioned three mechanical properties of blood samples effectively, the air cavity in the blood sample syringe must be minimized (*V_air, B_* = 0). However, it will be necessary to secure the air cavity in the reference fluid syringe (*V_air, R_* = 0.1 mL) for stabilizing fluidic instability resulting from the syringe pump.
